# Insights into the Regulatory Role of m^6^A Epitranscriptome in Glioblastoma

**DOI:** 10.3390/ijms21082816

**Published:** 2020-04-17

**Authors:** Silvia Galardi, Alessandro Michienzi, Silvia Anna Ciafrè

**Affiliations:** Department of Biomedicine and Prevention, University of Rome Tor Vergata, Via Montpellier, 1 00133 Rome, Italy; alessandro.michienzi@uniroma2.it

**Keywords:** N^6^-methyladenosine, glioblastoma, epitranscriptome, METTL3, METTL14, ALKBH5, FTO, YTHDF2, WTAP, NSUN5

## Abstract

N^6^-methyladenosine (m^6^A) is one of the most widespread and abundant internal messenger RNA modifications found in eukaryotes. Emerging evidence suggests that this modification is strongly linked to the activation and inhibition of cancer pathways and is associated with prognostically significant tumour subtypes. The present review describes the dynamic nature of m^6^A regulator enzymes, as methyltransferases, demethylases and m^6^A binding proteins, and points out thevalue of the balance among these proteins in regulating gene expression, cell metabolism and cancer development. The main focus of this review is on the roles of m^6^A modification in glioblastoma, the most aggressive and invariably lethal brain tumour. Although the study of m^6^A in glioblastoma is a young one, and papers in this field can yield divergent conclusions, the results collected so far clearly demonstrate that modulation of mRNA m^6^A levels impacts multiple aspects of this tumour, including growth, glioma stem cells self-renewal, and tumorigenesis, suggesting that mRNA m^6^A modification may serve as a promising target for glioblastoma therapy. We also present recent data about another type of epitranscriptomic modification, the methylation of cytosine at a specific site of 28S rRNA, as it was recently shown to affect the biology of glioma cells, with high potential of clinical implications.

## 1. Introduction

### 1.1. m^6^A Methylation

The last decade has yielded remarkable progress toward understanding how chemical modifications on RNA can influence cell biology. To date, thanks to the development of advanced chemical and/or biochemical methods, almost 170 types of RNA modifications have been identified and are globally termed as “epitranscriptome” [1]. Epitranscriptomic modifications embed transcripts with information additional to that carried in their sequence of bases, they can change the charge of RNA bases, alter their base-pairing properties, resulting in differential RNA folding, and form recognition elements that modulate protein–RNA interactions. Therefore, these modifications induce changes in RNA metabolism and are involved in the control of the delicate gene expression patterns that influence cell biology.

N^6^-methyladenosine (m^6^A) is the most widespread and abundant internal messenger RNA modification found in eukaryotes (m^6^A/A ratio in human polyadenylated RNA is estimated to be 0.4–0.7%), as well as in RNA of nuclear-replicating viruses [2]. Since m^6^A was first discovered in mRNA decades ago, only a few sites were mapped in cellular and viral RNAs, none of them in human, both in coding and non-coding regions. In 2012, to gain further insight into the role of m^6^A in RNA metabolism, Dominissini and co-authors [3] developed m^6^A-seq technique, combining the high specificity of an anti-m^6^A antibody applied to randomly fragmented transcripts with the power of massively parallel sequencing. Using m^6^A-seq, they compiled the first human and mouse RNA methylomes and demonstrated their evolutionary conservation and response to changing cellular conditions. Sequence analysis revealed that m^6^A modification generally occurs in the consensus motifRRACH (in which Rdenotes G or A; and Hdenotes A, C or U), where A is converted to m^6^A.Whereas RRACH motifs are rather frequent in mRNA, only a fraction of those sites are methylatedin vivo. How individual transcripts and specific sites are selected for proper deposition of m^6^A modification remains unclear. Notably, m^6^A methylation is nonrandomly distributed throughout the mRNA body, being enriched proximal to the stop codon (within both the coding DNA sequence (CDS) and the 3′ untranslated region (3′UTR)), and to a lesser extent within the 5′ untranslated region (5′UTR). Finally, m^6^A mapping identified specific gene categories that are associated with transcripts containing a disproportionately high level of m^6^A. Analysis of mRNAs with large numbers of mapped m^6^A sites revealed an enrichment for genes that regulate development and cell fate specification [3]. In contrast, highly stable transcripts encoding ‘housekeeping’ proteins, such as ribosomal proteins, were found to be reduced in m^6^A [4]. To date, more than 500 m^6^A-mapping studies have been deposited in gene expression databases; however, the exact stoichiometry of specific m^6^A sites and whether and how it changes in different conditions are not known.

### 1.2. m^6^A Regulators

#### 1.2.1. Writers

The m^6^A deposition is catalyzed by an RNA methyltransferase complex (MTC) also known as m^6^A ‘writer’; it occurs at the pre-mRNA level during transcription and mRNA processing and is completed by the time an mRNA is released from chromatin into the nucleoplasmic RNA [5]. The core component of MTC is a heterodimer composed of methyltransferase-like 3 (METTL3) and methyltransferase-like 14 (METTL14). METTL3 is the catalytic component and requires bound donor substrate S-adenosyl-methionine (SAM) in the catalytic site, while METTL14 is an inactive methyltransferase that serves as an allosteric activator of the enzymatic activity of METTL3, and also contributes to RNA binding of the complex [5]. A recent paper reported that m^6^A modifications are enriched in the vicinity of histone H3 trimethylation at Lys36 (H3K36me3) peaks, and are reduced globally when cellular H3K36me3 is depleted. H3K36me3, a marker for transcription elongation usually enriched in the CDS and near the 3′ end, is recognized and bound directly by METTL14 which recruits other components of the m^6^A MTC and mediates deposition of m^6^A on the newly synthesized RNAs when encountering RNA Pol II [6]. This study uncovered a mechanism that underlies the dynamic, specific and co-transcriptional deposition of m^6^A modification in mammalian transcriptomes, explaining its prevalent occurrence in the CDS and 3′UTR. The identification of the histone mark H3K36me3 as a determinant of m^6^A RNA modification via METTL14 reveals a crosstalk between histone modification and RNA methylation. The third component of the writer complex is Wilms’tumor 1-associating protein (WTAP), which cooperates with its cofactors KIAA1429 (VIRMA), ZC3H13, and RBM15/RBM15B, to anchor MTC in nuclear speckles and to confer specificity to m^6^A deposition in select groups of transcripts and sites therein [7,8].

Recently, METTL16 has been identified as another catalytically active m^6^A mRNA methyltransferase [9]. METTL16 is similar to METTL3 in structure but functions alone, relying on a combination of RNA sequence and structure to recognize its RNA substrates. METTL16-dependent m^6^A marks are found in introns, at intron–exon boundaries and on U6 small nuclear RNA that base-pairs with 5′splice sites of pre-mRNAs during splicing. These findings suggest that METTL16 plays an important role in mRNA stability and splicing [9].

#### 1.2.2. Erasers

The discovery of the m^6^A demethylase “fat mass and obesity-associated protein” (FTO) [10] and later of ALKBH5 [11], encouraged the hypothesis that m^6^A is dynamically modulated within cells, or across conditions, through active demethylation [12]. These two demethylases, named m^6^A “erasers” are supposed to ensure a balanced equilibrium of m^6^A modification in the transcriptome. However, multiple recent reports have hinted that m^6^A may not be a physiologically relevant target of FTO. For example, m^6^A levels were not increased in mRNA derived from *FTO* knockout mouse embryos and cells, and a transcriptome-wide mapping of m^6^A stoichiometry using Mazter-Seq also demonstrated that m^6^A stoichiometries throughout the transcriptome are not affected by FTO overexpression or depletion [11,13]. A progress in understanding the function of FTO was the discovery that FTO has mainly higher catalytic activity for demethylating m^6^Am (*N*6,2′-*O*-dimethyladenosine) than for m^6^A [14]. This, together with the difficulty of specifically detecting m^6^A vs. m^6^Am, might explain some apparently contrasting results about the effects of FTO depletion or reduction in several contexts. 

The Mazter-Seq method mentioned above, demonstrated that, when ALKBH5 is overexpressed, there is a subtle but reproducible decrease in methylation levels, increasingly pronounced at higher-confidence sites. ALKBH5 was shown to colocalize with nuclear speckles to regulate mRNA processing factors’ assembly/modification, and to modulate mRNA export and stability. In ALKBH5-deficient cells, cytoplasmic RNA level is significantly increased due to accelerated nuclear RNA export, nascent RNA synthesis is enhanced, global RNA stability is reduced [11,15]. ALBKH5 was shown to play an essential role in spermatogenesis [11]. Male mice lacking *Alkbh5* are completely infertile due to severe germ cell depletion [11]. Tang and co-authorsdemonstrated that ALKBH5-dependent m^6^A mainly controls mRNA fate in spermiogenesis. Appropriate m^6^A erasure is required for correct splicing of longer 3′-UTR transcripts in the nucleus, and m^6^A enrichment in 3′-UTRs of mRNAs correlates with enhanced degradation in the cytoplasm. ALKBH5-dependent m^6^A isrequired for meiotic and haploid phases of spermatogenesis by controlling both splicing and stability of mRNAs [16].

#### 1.2.3. Readers

The regulatory role of m^6^A on RNA molecules is similar to that of epigenetic marks on chromatin, which could be achieved in either *cis* or *trans ways*. In the *cis* mode, the effect of m^6^A on the RNA structure is similar to that of epigenetic marks on the nucleosome. Incorporation of the methyl group on the N6 position of adenosine impairs the stability of Watson–Crick A:U base-pairing, leading to further global conformational rearrangement of the RNAsubstrate [17]. Alternatively, m^6^A can mediate RNA functions in a *trans* mode through the recruitment of specific proteins or protein complexes, known as m^6^A “readers”. One of the first identified and characterized reader protein family is the YT-Homology(YTH)-domain-containing protein family [6,18]. By targeting different complexes to specific sites via direct binding to m^6^A, the YTH-domain-containing proteins participate extensively in post*-*transcriptional regulation by regulating splicing, translation, localization, and lifetime of RNAs [19]. There are five YTH-domain-containing proteins in humans, namely, YTHDF1, YTHDF2, YTHDF3, YTHDC1, and YTHDC2. YTHDF2 is the first protein, of which the m^6^A-associated function has been well studied. After being targeted to a specific site via m^6^A recognition, YTHDF2 recruits the CCR4-NOT deadenylase complex to destabilize and further decay target mRNAs [6,20]. Binding of YTHDF1 to m^6^A-modified mRNA increases the translation efficiency of the mRNA independent of the m^7^G cap [18]. YTHDC1, the only known m^6^A reader in the nucleus, has been reported to be involved in exon selection during splicing [19], epigenetic silencing mediated by the noncoding RNA XIST [21], and the nuclear export of mRNA [22]. Unlike the other YTH proteins, which are ubiquitously expressed, YTHDC2 is enriched in the testis. It is a putative RNA helicase that forms a complex with the meiosis-specific coiled-coil domain-containing protein (MEIOC) to regulate RNA levels during meiosis and promoting translation [23].

Additional putative direct m^6^A readers with common RNA-binding domains but without a YTH domain, have recently been discovered. For instance, the m^6^A reader heterogeneous nuclear ribonucleoprotein C (HNRNPC), an abundant nuclear RNA-binding protein responsible for pre-mRNA processing including splicing, was shown to bind m^6^A-modified RNA through a “m^6^A-switch” mechanism, in which the m^6^A-mediated destabilization of an RNA hairpin exposes a single-stranded HNRNPC binding motif [24]. Additional heterogeneous nuclear ribonucleoproteins as HNRNPA2B1 and HNRNPG were shown to influence a large number of alternative splicing events in an m^6^A-dependent manner. In particular, HNRNPA2B1 directly binds a set of nuclear transcripts, eliciting similar alternative splicing effects as the m^6^A writer METTL3, and also regulates pri-miRNA processing [25]. The eukaryotic initiation factor 3 (EIF3) was shown to promote cap-independent translation, through the direct binding to m^6^A residues in the 5′ UTRs. Thus, the presence of m^6^A bypasses the normal requirement for eIF4E and may be a mode of translation that is important when eIF4E (the 7-methylguanosine-containing mRNA cap-binding protein) function is impaired. Because only a few mRNAs contain m^6^A in the 5′ UTR, this mechanism is limited to a small subset of the m^6^A-containing mRNAs [26]. Also, ribosomes may function as m^6^A readers. Studies using single-molecule ribosome translocation assays showed that mammalian ribosomes have a tendency to stall on mRNA at m^6^A-containing codons. The extent to which this mechanism affects m^6^A mRNA stability and/or translation remains unclear [27].

Interestingly, at least two “antireaders” of m^6^A have been identified. RasGTPase-activating protein-binding protein 1 (G3BP1) and 2 (G3BP2), that function in the assembly of stress granules and are known to be important for embryonic development [28], are repelled by the presence of m^6^A. The target sequences of G3BPs within RNAs substantially overlap with the binding sites of YTHDF2 and YTHDC1; however, G3BP1 and G3BP2 preferentially bind unmethylated sequences [28,29]. 

### 1.3. m^6^A in Human Cancer

Emerging evidence suggests that m^6^A modification is involved in human carcinogenesis. A recent systematic analysis (> 10,000 patients across 33 cancer types) of the landscape of molecular alterations and clinical relevance of m^6^A writers, erasers and readers demonstrated that these regulators are strongly linked to the activation and inhibition of cancer pathways and are associated with prognostically significant tumour subtypes [30]. These alterations have been implicated to play a role in different cancer functions, including cancer stem cell formation, epithelial–mesenchymal transition (EMT), cancer metabolism, apoptosisand signal transduction, by regulating the mRNA stability or protein translation of different downstream targets. METTL3 was shown to associate with ribosomes, and to promote translation of oncogenic mRNAs, including EGFR and the Hippo pathway effector TAZ, leading to lung and colon cancer cell growth, survival, and invasion [31]. METTL3 mRNA and protein are overexpressed in acute myeloid leukemia cells, compared with healthy hematopoietic stem/progenitor cells [32,33]. METTL3 associates with chromatin and localizes to the transcriptional start sites of active genes and induces m^6^A modification within the coding region of the associated mRNAs, including c-Myc and BCL2 mRNAs, enhancing their translation by relieving ribosome stalling [32]. Consequently, METTL3 inhibits hematopoietic stem/progenitor cell differentiation and increases leukemia cell growth, and METTL3 depletion induces cell-cycle arrest, cell differentiation, and apoptosis and delays leukemia progression in mice.

METTL3 was shown to impair also the EMT of cancer cells [31]: m^6^A-sequencing and functional studies confirmed that Snail, a key transcription factor of EMT, is involved in m^6^A-regulated EMT. m^6^A in Snail CDS, but not 3′UTR, triggers polysome-mediated translation of Snail mRNA in cancer cells. Loss and gain functional studies confirmed that the reader YTHDF1 mediates m^6^A-increased translation of Snail mRNA [31]. Another recent report identified a novel mechanism involving the translation initiation factor EIF3C, as the direct target of the reader YTHDF1 [34]. YTHDF1 augments the translation of EIF3C by binding to m^6^A modified EIF3C mRNA and concomitantly promotes the overall translational output. Global translation rates are generally enhanced in cancer cells and altered translational control is a fundamental response to oncogenic stimulation.m^6^A methylome analysis also identified that multiple translation initiation factors were modulated by m^6^A, indicating that m^6^A modification could directly regulate the expression of translation associated factors, thereby facilitating tumorigenesis and metastasis of ovarian cancer [34]. The expression of YTHDF1 was found to be induced by the oncogenic transcription factorc-MYC, and this axis c-MYC/YTHDF1 promotes colon cancer cell proliferation and induces the resistance against anticancer drugs [35]. Another reader, YTHDF2, was found to bind c-Myc and CEBPA mRNAs to enable m^6^A modification in the 5′-UTR and CDS, promoting c-Myc and CEBPA mRNA stabilization and leukemia cell proliferation [36]. In addition, YTHDC2 upregulates the expression of metastasis-related proteins such as HIF-1alpha, by promoting mRNA translation, and thereby induces colon cancer metastasis [37].

### 1.4. m^6^A in Glioblastoma

The study of m^6^A epitranscriptome in glioma is a young one, as papers in this field started to be published only three years ago. Intriguingly, among them, about one half seem to support a general reduction of m^6^A methylation levels in this tumour, indicating a protective role for this modification, while the other half sustain the opposite view.

#### 1.4.1. Reports About Reduced m^6^A RNA Methylation in Glioblastoma

Li et al. very recently reported that m^6^A RNA methylation is reduced in glioma tissues, and that ectopically increasing m^6^A levels by METTL3 overexpression in one glioma cell line could impair its proliferation and migratory ability, while increasing apoptosis [38]. However, that work did not dig into the mechanism through which this epitranscriptomic modification may affect glioblastoma growth. 

The first paper soundly addressing this point and describing the involvement of m^6^A RNA methylation and of m^6^A-related proteins in glioblastoma was published in 2017, by Shi’s group [39]. The model they chose were glioblastoma stem cells (GSCs), considered the initiating cells of glioblastoma, usually enriched in restricted niches and deemed responsible not only for glioblastoma onset but also for its resistance to therapy and eventual recurrence [40]. 

When GSCs were induced to differentiate in vitro, Cui and co-authors observed an increase in m^6^A RNA methylation [39]. In parallel, the knock-down of two key m^6^A “writers”, METTL3 and METTL14, while reducing general m^6^A methylation, enhanced the GSC proliferation and self-renewal ability, and in turn METTL3 overexpression reduced the same tumorigenic properties of GSCs. These results, suggesting an inverse correlation between GSC tumorigenicity and the general levels of m^6^A RNA methylation, were further corroborated by in vivo data, showing that the knock-down of METTL3 or METTL14, or both, significantly enhanced the growth of GSC-generated tumours. These authors also provided clinically relevant data showing that MA2, the ethyl ester form of meclofenamic acid (MA), and a selective inhibitor of FTO, boosted m^6^A levels, reduced GSC growth and self-renewal in vitro, and severely impaired GSC-induced tumorigenesis in vivo. Mechanistically, by combining m^6^A-seq analysis in GSCs, and RNA-seq in GSCs depleted of METTL3 or METTL14, the authors revealed key mediators of the functional effects of m^6^A RNA methylation perturbation in glioblastoma initiating cells. Among them, several oncogenes, whose expression increased upon METTL3 or METTL14 depletion, and was reduced by MA2 treatment, in an inverse correlation with the levels of specific m^6^A methylation of their mRNAs. 

Almost at the same time as Cui and colleagues’ publication, Huang’s group gave its important contribution to the concept of m^6^A RNA methylation importance in glioblastoma [41]. Their work showed that the demethylase ALKBH5 is highly expressed in GSCs, and, notably, overexpressed in those cells not only in comparison with healthy control tissues or cells, but also compared to established glioblastoma cell lines. Moreover, ALKBH5 mRNA expression resulted as a negative prognostic factor for glioblastoma patients, whereby a low ALKBH5 mRNA expression correlated with a longer overall survival. In agreement with the enrichment of ALKBH5 expression in GSCs, the protein colocalized, in tumour tissues, with two typical stemness markers for glioblastoma, SOX2 and Nestin. The comparison between glioblastoma stem cells and tumour tissues derived from the same patient evidenced ALKBH5 mRNA strong enrichment in the stem cells, again pointing at a role for ALKBH5 not merely in glioblastoma, but rather in its cells of origin. In fact, the depletion of ALKBH5 in GSCs reduced their self-renewal and proliferation, and impaired their ability to form a tumour. 

Even if the authors indeed showed an overall increase of m^6^A levels in ALKBH5-depleted cells, they could strongly restrict the number of the genes affected by ALKBH5 depletion—both from the RNA methylation and the expression level point of view—causatively involved in the impaired tumorigenicity of GSCs. Among them, the authors focused on FOXM1, a pivotal transcription factor in cell-cycle regulation, self-renewal and tumorigenesis of GSCs [42], as its mRNA was hypermethylated upon depletion of ALKBH5, and its expression, at both mRNA and protein levels, was reduced by ALKBH5 knock-down. They went much deeper in the mechanism, by demonstrating that a unique methylation site in FOXM1 mRNA 3′UTR affected by ALKBH5 is responsible for ALKBH5 effects on FOXM1 expression, as the methylation at that site impairs binding of the nuclear RNA binding protein HuR to FOXM1 nascent mRNA. As HuR interaction with FOXM1 mRNA promotes its expression, the site-specific hypermethylation of FOXM1 3′UTR upon ALKBH5 depletion results in FOXM1 reduction. To confer even more specificity to this mechanism, the authors discovered that in GSCs, the long noncoding RNA antisense to FOXM1, FOXM1-AS, facilitates the interaction of ALKBH5 with FOXM1 nascent RNA, inducing demethylation, in turn increasing HuR binding and FOXM1 expression. 

Such an intricate and elegantly demonstrated regulatory network affecting the cells of origin of glioblastoma is pivotal to highlight how specific can be the role of one general regulator of m^6^A methylation in a specific context, and, as a consequence, how difficult and “risky” it may be to draw functional conclusions by the sole observation of the differential expression of single members of m^6^A epitranscriptome operating protein family, or the measurement of the general level of m^6^A.

While our manuscript was in preparation, a paper was published reporting the possibility of impairing the in vitro migration and invasiveness of the U87MG glioblastoma cell line, by the supplementation of MV1035, an inhibitor of ALKBH5 [43]. This result supports the role of ALKBH5 as a positive regulator of glioblastoma tumorigenic properties.

Among gliomas, somatic mutations in the genes IDH1 and 2 occur in about 80% of grade II-III gliomas and secondary glioblastoma (GBM), while they are very rare in grade IV glioblastomas [44]. In fact, IDH1 and IDH2 mutations correlate with a better overall survival of glioma patients. IDH genes encode for isocitrate dehydrogenase 1 and 2, which catalyze the oxidative decarboxylation of isocitrate to α-ketoglutarate (α-KG) in an NADP+-dependent manner. The somatic mutations frequently present in low-grade gliomas induce a neomorphic enzymatic function able to catalyze the conversion of α-KG to R-hydroxyglutarate (R-2HG) [45]. The apparent conundrum of mutations specifically arising in tumours but representing good prognostic factors compared to the wild type forms of the same genes was addressed by Su R. and collaborators in a huge work essentially devoted to leukemia oncogenesis, but with important implications for glioma too [36. What they found is tightly related to m^6^A methylation of RNA, and in particular to one key demethylase, FTO. They showed that R-2HG inhibits FTO, in turn increasing the overall levels of m^6^A methylation. One of the key mRNA targets whose methylation changed upon R-2HG administration in IDH1 or 2 wild type leukemia cells was the MYC one, thus made less stable, with a consequent reduction of Myc expression and signalling. The downstream functional effects of R-2HG administration in IDH1 or 2 wild type cells were all anti-tumour ones, and the results of the experiments had also important implications regarding the possibility of combining R-2HG with standard chemotherapy in IDH1/2 wild type tumours. Even if this work was mostly performed in leukemia cells, and all mechanistic demonstrations were made in that context, the authors also showed that R-2HG can inhibit the proliferation of a panel of glioma cell lines, all IDH1/2 wild type. They suggested that the endogenous production of R-2HG typical of low-grade gliomas may prevent the progression of such tumours toward higher grade glioblastomas. Of course, a corollary of their observation should be that, in low grade, IDH1/2 mutant gliomas, the levels of FTO, kept low by R-2HG, result in an overall high level of m^6^A (or likely m^6^Am) RNA methylation, particularly relevant on MYC transcripts. In turn, this should mean that, in glioma just as in leukemia, tumours with high FTO levels and low/medium MYC levels should be the most sensitive ones to R-2HG treatment. Dedicated studies are needed to understand if this is indeed the case, and this is a path which deserves to be walked to search for yet unknown possible vulnerabilities of glioma cells.

#### 1.4.2. Reports About Increased m^6^A RNA Methylation in Glioblastoma

As early as in 2016, the Liu’s group published a paper showing that WTAP expression predicts poor prognosis in malignant glioma patients [46]. As WTAP is a crucial interactor of the methyltransferase complex, this and other observational works [47] suggested that m^6^A inducing enzymes, and, as a consequence, m^6^A RNA methylation, may play an oncogenic role in glioma.

However, the first mechanistic work linking m^6^A methylation and oncogenesis in glioblastoma was published almost two years later, by Visvanathan and co-authors, who studied the levels of m^6^A RNA methylation in three glioblastoma stem cell lines and showed that they were reduced uponin vitro differentiation [48]. Moreover, they observed that METTL3 mRNA was clearly more abundant in GSCs compared to their differentiated counterparts. By knocking-down METTL3 in GSCs, they obtained a significant reduction of self-renewal and of the expression of GSC-specific markers, while the fraction of live cells was reduced due to an increase in the apoptotic rate.

Mechanistically, they identified SOX2 mRNA, encoding for a key stemness marker for GSCs, as a target for METTL3 function. SOX2 mRNA, in fact, was directly bound by METTL3, and its m^6^A methylation levels were diminished upon silencing of METTL3. In METTL3-silenced GSCs, Sox2 expression was reduced at both the mRNA and the protein level. The authors showed that three sites, located in SOX2 mRNA 3′UTR, and matching the METTL3 consensus responsive element GGACH, are recognized by METTL3 and affected by m^6^A methylation; the sequence integrity of all three sites is needed for METTL3 binding and methylation [48]. 

The clue linking SOX2 mRNA m^6^A methylation and stabilization resides in the RNA binding protein HuR. As demonstrated for FOXM1 nascent RNA [38], HuR was shown to bind and stabilize SOX2 mRNA. However, in contrast to what described for FOXM1, in this paper the authors showed that m^6^A RNA methylation by METTL3 is necessary for HuR binding to SOX2 mRNA and for its stabilization. This result was further extended to the total pool of RNAs bound by HuR, whichwas strongly reduced in the absence of METTL3. It is necessary to highlight that here the authors did not claim the existence of a precise overlap of METTL3 and HuR binding sites along SOX2 and other target mRNAs, differently from FOXM1. Rather, they reported a “proximity” of recognition sites in the range of ±50 nt.

An added value of Visvanathan’s work is the analysis of m^6^A RNA methylation role in one of the crucial features of glioblastoma cancer stem cells, i.e., their resistance to radiotherapy. Indeed, these authors observed that METTL3-depleted GSCs were more sensitive to γ-irradiation, and specifically showed that SOX2 mediates at least in part the METTL3-dependent radioresistance of GSCs, by inducing the homologous recombination DNA repair response. Of clinical relevance is also the result about the reduced in vivotumorigenicity of U87 stem-like cells depleted of METTL3, together with the evidence of METTL3 mRNA and protein higher abundance in glioblastoma tissues compared to normal brain.

The same group went further in its study of METTL3 role in glioblastoma stem cells by performing an integrated analysis of m^6^A-RIP (RNA immunoprecipitation) and total RNA-Seq of METTL3-silenced GSCs [49]. In their work, in great part based on in silico analyses of the sequencing data obtained in one GSC line silenced for METTL3, they showed that m^6^A methylation was strongly impaired in METTL3 knocked-down cells, thus confirming the primary importance of this enzyme for global m^6^A RNA methylation in GSCs. They also found an enrichment of METTL3-affected m^6^A sites around the stop codon and in the 3′UTR of mRNAs, in agreement with other studies. The knock-down of METTL3 produced a widespread downregulation of protein coding gene expression, in particular of those genes whose mRNAs contained METTL3-mediated m^6^A sites. However, many other indirect METTL3 targets were found to be modulated, and mostly downregulated, upon METTL3 depletion, indicating that METTL3 may work by positively regulating its direct targets, which in turn regulate a wider pool of transcripts. One key example of a transcript which is a direct target of METTL3 and whose abundance is reduced in METTL3 knock-down cells is SOX2, as previously demonstrated by the same group. In addition, a number of other GSC transcription factors were repressed upon METTL3 deprivation, even if their mRNAs were not affected by m^6^A methylation, indicating, again, that the METTL3 tumoral role in GSCs may go beyond the pool of mRNAs directly affected by its enzymatic activity.

Several aspects of RNA metabolism were addressed by the authors, who in fact could show a drastic reduction of A to I RNA editing events in exonic regions, likely due to a direct, m^6^A methylation-mediated, regulation of the editing enzymes ADAR and ADARB1 by METTL3. In addition, they showed that METTL3 knock-down produced a wide perturbation of splicing in GSCs, such as intron retention and exon inclusion, and novel 5′ and 3′ alternative splicing sites. Among these aberrations though, only the exon skipping reduction observed in METTL3 KD cells appeared to be directly linked to m^6^A RNA methylation of the respective transcripts. Thus, as for general gene expression results, also these specific aspects of RNA stability and processing appear to be linked to m^6^A methylation in two alternative ways, either directly, or indirectly.

A further peculiarity of this work was that it analyzed the effects of METTL3 silencing not only on protein coding RNAs, but also on noncoding ones. In particular, Visvanathan and colleagues measured that METTL3 depletion induced a generalized increase in lincRNA expression (opposite to what was observed for mRNAs). In apparent contradiction with this, they observed that the most highly expressed lincRNAs were those with the highest levels of m^6^A methylation, and were those whose expression was instead reduced upon METTL3 depletion, suggesting, again and as for coding RNAs, that m^6^A methylation contributes to the stabilization of transcripts. Thus, METTL3 depletion had a general enhancing effect on the basic level of lincRNAs, but a specific, opposite and repressing effect on those lncRNAs which are normally the most abundant ones, and likely the most relevant ones.

The large amount of data collected and analyzed in this paper holds a great interest for the comprehension of METTL3-mediated m^6^A methylation in glioblastoma, even if a limitation of this study is that almost all analyses are in silico ones, based on a single GSC line. 

A specific and pervasively important oncogenic role of METTL3 was described by Li and co-authors [50], who showed that METTL3 modulates the nonsense-mediated RNA decay (NMD) of splicing factors mRNAs, and, as a consequence, can induce extensive alternative splicing isoform switches able to deeply perturb the metabolism of glioblastoma cells.

After showing, by comparing previously deposited MeRIP-seq data of one glioma stem cell line and neural progenitor cells, that the two cell types were clearly distinct from the point of view of m^6^A RNA methylation, these authors also evidenced a significantly higher expression of METTL3 and YTHDF2, at both the mRNA and the protein levels, in GBM patients’ samples compared to normal brain controls, and highlighted that METTL3 and YTHDF2 expression negatively correlated with patient survival.

In both established glioblastoma cell lines and in three different primary cell lines derived from patients, the CRISPR/Cas9-mediated depletion of METTL3 as well as the overexpression of a catalytically inactive dominant negative form of METTL3 in vitrostrongly reduced the proliferation, migration and invasiveness, while increasing the apoptotic index of U87 cells, and in vivo reduced the tumour formation rate. Thus, the methylation activity of METTL3 is critical for the oncogenic role of this protein in glioblastoma.

The mechanism explaining this phenotype was investigated by Li and colleagues, who found, in partial agreement with Visvanathan and colleagues [49], that METTL3 depletion in the U87 cell line induced the downregulation of the splicing factors SRSF3, SRSF6, and SRSF11. In turn, the reduction of these factors reduced the proliferation of glioblastoma cell lines. Of particular interest, the reduction of mRNAs encoding for these splicing factors was due to an increase of the non-protein-coding isoforms of the same mRNAs, in turn due to aberrant splicing leading to nonsense-mediated decay (NMD).

The link to m^6^A RNA methylation was found in the specific m^6^A methylation sites around the start codons of SRSF mRNAs: by combining a sophisticated reporter system and different single point mutations at the methylated adenosines of SRSF6 and SRSF3, the METTL3-mediated m^6^A RNA methylation was shown to be necessary for inhibiting the NMD of SRSF mRNAs. A step forward was taken by this group, which identified also the m^6^A reader responsible for this mechanism: YTHDC1 was shown to bind the m^6^A residues around SRSF start codons, and YTHDC1 knock-down induced the NMD, together with the reduction of in vitro tumorigenic properties of U87 cells.

The ultimate result downstream of this is the comprehension of a widespread perturbation of alternative splicing in turn depending on m^6^A RNA methylation and NMD of SRSF mRNAs: (i) m^6^A RNA methylation increases the amount of SRSFs thanks to the recognition of methylated adenosines in SRSF mRNAs by YTHDC1, thus counteracting the physiological levels of NMD of these transcripts, which in healthy cells buffer SRSF amounts; (ii) the enhanced expression of SRSFs modulates alternative splicing in a GBM-specific pattern, with a shift toward exon inclusion in several mRNAs encoding for cancer relevant factors, such as the antiapoptotic transcript variant of Bcl-X and the GSC-promoting isoform of NCOR2.

This work holds an undeniable value sustaining the protumoral role of METTL3-mediated mRNA methylation in glioblastoma. However, it must be underlined that its mechanistic part, involving SRSFs, YTHDC1, alternative splicing, and Bcl-X and NCOR2 isoforms, relies on experiments performed in a single established cell line, U87. More extensive results are needed, possibly obtained in GSCs, to understand the general significance of the proposed model.

Recently, Chai and co-authors performed an observational study aimed at understanding if the expression of factors involved in m^6^A (writers, erasers, readers) can be used in a purely prognostic perspective for glioma [51]. They correlated the mRNA expression levels of 13 m^6^A methylation regulators with the clinicopathological features of gliomas, by deriving the data from those deposited in The Cancer Genome Atlas (TCGA) [52] and the Chinese Glioma Genome Atlas (CGGA) [53] datasets. They reported a direct correlation between the expression of WTAP and RBM15, both components of the methyltransferase complex, ALBKH5, an eraser, YTHDF2, a reader, and the WHO grade, while an inverse correlation was found with the expression of the eraser FTO. In general, they did not find any statistically significant correlation between METTL3, METTL14 or METTL16 mRNA expression and the WHO grade.

As for the prognostic value of these results, by performing a univariate Cox regression analysis on the expression levels in the CGGA dataset, these authors found that eleven out of thirteen tested genes were significantly correlated with OS, with in particular FTO behaving always as a protective gene in all types of glioma, including low-grade gliomas and glioblastomas, both IDH wild-type and IDH mutant. They could further restrict the list of predictive genes to a seven-gene signature, including FTO and YTHDC1 as “protective” genes, and YTHDF1, YTHDF2, ALBKH5, RBM15 and WTAP as “risky” genes. Based on this signature, glioma patients of the CGGA and the TCGA cohorts could be separated into low- and a high-risk groups; notably, the OS of high-risk groups was significantly lower than the low-risk ones, in both the TCGA and the CGGA dataset. Most importantly, the seven-gene signature could independently predict the prognosis of grade II, grade III, and grade IV glioma patients, whereby patients with high risk scores had significantly shorter overall survival than those with low risk score. Furthermore, and of clinical importance, the highrisk score patients were more sensitive to temozolomide, suggesting the possibility of using this signature to set-up the treatment protocol.

### 1.5. Not Only m^6^A Methylation, Not Only mRNA. The Case of 5-Methylcytosine in rRNA

Adenosine is not the only nucleotide in RNA that can undergo post-transcriptional methylation. Among others, cytosine too can be turned into 5-methyl-cytosine by the action of the RNA cytosine methyl-transferases NSUNs. This epitranscriptomic modification can affect tRNA, mRNA, and rRNA molecules where it occurs [54].

Only one relevant paper was recently published about the involvement of this type of modification hitting 28S rRNA in glioma [55], and the findings described therein hold very interesting aspects with potential clinical implications for glioma patients. 

Starting from the evidence of the epigenetic silencing of the promoter of the NSUN5 gene through the hypermethylation of a CpG island in several glioma cell lines, correlating with NSUN5 transcriptional silencing, this group showed that NSUN5 behaves as a tumour suppressor in several in vivo mouse models of glioma growth.

The authors of this paper identified the C3782 position of human 28S rRNA as the human target for NSUN5. This site is in close vicinity with the peptidyltransferase centre of the large ribosomal subunit, and the authors found that its unmethylated form may change the conformational state of the P-site in the ribosome.By using different experimental approaches, they showed that indeed the removal of the methyl group at C3782 can impair normal protein synthesis by altering the P-site.

The ultimate result of their work showed thatin glioma cells, the epigenetic loss of NSUN5, in a frame of general restriction of the protein synthesis, induces the selective synthesis of specific proteins. By matching the proteins upregulated in NSUN5-silenced cells with the mRNAs whose translation was increased in the same conditions, they found a group of stress-related proteins, and focused on the stress-activated protein NQO1, a multifunctional antioxidant enzyme [56]. Interestingly, they showed that NSUN5-silenced glioma cells are far more sensitive to drugs targeting NQO1 than cell lines where NSUN5 is expressed, and this was reflected also in vivo, where specific anti-NQO1 drugs could increase the survival of mice orthotopically injected with NSUN5-deficient glioma cells, but did not influence the fate of animals injected with NSUN5 proficient cells.

All these observational and mechanistic studies were further corroborated by results endowed with very important clinical significance. The analysis of the data present in the TCGA database led to discover that: (i) 28% of the annotated gliomas shows CpG island hypermethylation in NSUN5 promoter, correlating with transcript downregulation, (ii) this condition is particularly enriched in low-grade gliomas compared to GBM, (iii) NSUN5 hypermethylation and low levels of expression positively correlate with longer overall survival in all glioma grades. Moreover, by studying a different validation cohort, the authors could confirm and extend these results, by calculating a positive correlation between NSUN5 hypermethylation and progression-free survival in both low grade gliomas and in glioblastomas. Of note, multivariate Cox regression analysis showed that NSUN5 hypermethylation could independently predict overall survival and progression-free survival in the TCGA and in the validation cohort, respectively.

The value of this work goes beyond the specific experimental results it shows, as it provides an additional proof, together with a new key molecular player, in favour of the so-called “translational paradox”, meaning that cells in stress conditions, while reducing the general rate of translation, can engage in a specific translational program in order to survive in those adverse conditions. Moreover, and specifically for glioma, this apparent paradox is tightly linked to another seemingly controversial observation of a condition—NSUN5 depletion via promoter hypermethylation—which favours tumour growth but at the same time is a good prognostic factor. As mentioned by the authors, this is true for IDH1/2 mutations too, and likely depicts a state where tumour cells struggle to deal with metabolic and hypoxic stress: by silencing NSUN5, they try their last choice for survival, reducing global protein synthesis but switching on emergency translational programs which include NQO1. However, this makes them more susceptible to specific therapies, thus contributing to the better prognosis of glioma patients characterized by NSUN5 silencing.

## 2. Discussion

It is striking that, among such a limited number of papers published so far about epitranscriptomic modifications in glioma, roughly one half indicate a protumoral role of m^6^A RNA methylation, whereas the remaining half sustain the opposite (Table 1). Thus, it is likely that the question itself—protumoralvs. tumour-suppressor role for m^6^A methylation—is inappropriate. As for almost all basic regulatory mechanisms, it is not the mechanism itself, or even a general perturbation of that mechanism, to be causatively linked to a pathological state, but rather the specific molecules affected by the dysregulated mechanism, embedded in a specific context. 

Not a secondary issue in this regard is the high heterogeneity of glioblastoma, which is obviously reflected in all in vitro models of this tumour. This might explain, at least in part, the contrasting results obtained by different groups in this field, as for example the correlation of m^6^A RNA methylation extent with differentiation of glioblastoma stem cells, or the effects of METTL3 knock-down on the in vitro self-renewal and in vivotumorigenicity of these cells (compare [39] with [48]). Glioblastoma stem cells, as well as the tumours from which they derive, can be classified into subtypes, characterized by different signatures and by different aggressiveness and responsiveness to therapies [57]. The subtypes of the GSCs used as models in the different papers discussed here were not always defined, and this simple issue might make a great difference. Moreover, all cited papers were, for obvious feasibility reasons, based on a very limited number of cellular models, where the effect of variability may be strongly felt. In our direct experience, for example, when we performed an RNA-seq analysis of seven different GSC lines, compared to healthy astrocytes, to normal human brain, and also to one established glioblastoma cell line, we did not find any evidence of a significant differential expression of any of the mRNAs encoding proteins involved in m^6^A methylation, from any side (i.e., writers, readers, erasers, our unpublished data).

However, the observed controversies do not weaken in any waythe results of those works produced by analyzing large datasets of clinical specimens, where the expression levels of some of the involved mRNAs are proposed as potential prognostic markers for glioblastoma. 

Regarding the mechanism though, it would be more appropriate to ask which mRNAs are hyper- or hypomethylated, in which specific context (e.g., GSCs or established tumours), and by which readers their methylation is read. In this regard, it may be interesting to mention that IGF2BPs were previously shown to play oncogenic roles in glioblastoma and specifically in GSCs, where their function as enhancers of the stability of specific target mRNAs was correlated to their ability to induce or sustain glioblastoma growth [58,59,60]. By combining these previous data with our present knowledge about IGF2BPs as m^6^A readers, we can get a deeper insight into an additional molecular mechanism through which these proteins may be involved in glioblastoma oncogenesis.

An additional very interesting result potentially linked to m^6^A methylation in glioblastoma was recently published by Wu and co-authors, who demonstrated that the m^6^A reader PRRC2a plays a key role in oligodendroglial specification and myelination by binding to a methylated adenosine in the coding region of OLIG2 mRNA [61]. This binding stabilizes OLIG2 mRNA and, as consequence, Olig2 protein production. On the contrary, the m^6^A eraser FTO demethylates OLIG2 mRNA at that site, thus promoting its degradation and Olig2 protein depletion. This work is clearly focused on oligodendrocyte determination in physiological and pathological hypomyelination conditions, but it is worth underlining that Olig2, besides its role as master factor in oligodendrocyte development, has also an important function in GBM cell reprogramming, genotoxic resistance, and tumor phenotype plasticity [62,63,64]. Moreover, its expression is a recognized marker of the proneural subtype of glioblastomas [57] and, even more importantly, in cooperation with Sox2, Pou3f2 and Sall2, Olig2 is a key master transcription factor of glioblastoma-initiating cells [62]. Thus, Prrc2a “reading” of OLIG2 mRNA (and its demethylation by FTO) might be relevant in the context of glioblastoma too.

This observation is only one example of how the recent knowledge about proteins involved in the epitranscritome modulation might be transferred to specific pathologic conditions, and to glioblastoma in particular, where this field surely deserves further and deeper studies.

The ultimate message we get from all these recent papers about epitranscriptomic modifications in glioma is that we are likely observing only the tip of the iceberg, and we will surely find many more sophisticated mechanisms which can finely modulate transcript fate in glioma cells. As the techniques employed to analyzeepitranscriptomic modifications become more and more refined [65,66], we become able to detect subtle changes even in little portions of the transcripts, as clearly shown in the case of the unique cytosine methylation event in 28S rRNA by NSUN5. As Janin’s paper teaches, a single, tiny change in one nucleotide of a single type of RNA can dramatically change a cell’s fate [55].

Moreover, one should never overlook that a crosstalk may exist between different epitranscriptomic modifiers affecting the same transcripts, as shown, for example, between the A to I RNA editing enzyme ADAR1 and m^6^A methylation. In specific transcripts, in fact, the association of ADAR1 was shown to be impaired by m^6^A methylation [67].

Our next task will be to understand if and how such tiny and dynamic changes may affect the pathological evolution of glioma.

## Figures and Tables

**Table 1:** Examples of tumour-suppressor vs. oncogenic roles of N^6^-methyladenosine (m^6^A) in glioblastoma, inferred from the study of specific factors involved in m^6^A modulation. NMD: nonsense-mediated decay; n.a.: not determined.

Factor Involved in m^6^A RNA Modification	Mediator/Target Molecules	Cell or Tissue Model	Inferred Tumorigenic vs Tumour-Suppressor Role of m^6^A RNA Methylation	Ref.
METTL3	n.a.	Glioma tissues and cell lines	Tumour-suppressor	[38]
METTL3 and METTL14	several oncogenes, whose expression increased upon METTL3 or METTL14 depletion	Glioblastoma stem cells	Tumour-suppressor	[39]
ALKBH5	FOXM1, HuR, FOXM1-AS	Glioblastoma stem cells and tissues	Tumour-suppressor	[41]
ALKBH5	n.a.	One glioblastoma cell line	Tumour-suppressor	[43]
FTO	MYC and other oncogenes	Glioma cell lines	Tumour- suppressor	[36]
WTAP	n.a.	Glioma tissues	Oncogenic	[46]
METTL3	SOX2, HuR	Glioblastoma stem cells	Oncogenic	[48]
METTL3	Several aspects of coding and noncoding RNA metabolism (splicing, editing)	One glioblastoma stem cell line	Oncogenic	[49]
METTL3 and YTHDC1	Modulation of NMD of mRNAs for splicing factors SRSF3, SRSF6, and SRSF11	Glioblastoma stem cells and U87 glioblastoma cell line	Oncogenic	[50]

## Abbreviations

VIRMA	Vir Like M6A Methyltransferase Associated
ZC3H13	Zinc Finger CCCH-Type Containing 13
RBM15	RNA Binding Motif Protein 15
RBM15B	RNA Binding Motif Protein 15 B
ALKBH5	AlkB Homolog 5, RNA Demethylase
TAZ	Tafazzin
BCL2	BCL2 Apoptosis Regulator
CEBPA	CCAAT Enhancer Binding Protein Alpha
FOXM1	Forkhead Box M1
HuR	ELAV Like RNA Binding Protein 1
SOX2	SRY-Box Transcription Factor 2
ADAR	Adenosine Deaminase RNA Specific
ADARB1	Adenosine Deaminase RNA Specific B1
Bcl-X	Apoptosis Regulator Bcl-X
NCOR2	Nuclear Receptor Corepressor 2
NQO1	NAD(P)H QuinoneDehydrogenase 1
IGF2BP	Insulin Like Growth Factor 2 MRNA Binding Protein
OLIG2	Oligodendrocyte Transcription Factor 2
POU3F2	POU Class 3 Homeobox 2
SALL2	Spalt Like Transcription Factor 2
PRRC2A	Proline Rich Coiled-Coil 2A
